# Pharmacological Evaluation of the Traditional Brazilian Medicinal Plant *Monteverdia ilicifolia* in Gastroesophageal Reflux Disease: Preliminary Results of a Randomized Double-Blind Controlled Clinical Trial

**DOI:** 10.3390/ph17111559

**Published:** 2024-11-20

**Authors:** Maitê Scherer da Silva, Rebeca Vargas Antunes Schunck, Maicon Pereira Moraes, Giana Blume Corssac, Gabriela Meirelles, Sara Elis Bianchi, Leonardo Vieira Targa, Valquiria Bassani, Marcelo Rodrigues Gonçalves, Caroline Dani, Ionara Rodrigues Siqueira

**Affiliations:** 1 Programa de Pós-Graduação em Ciências Biológicas—Farmacologia e Terapêutica, Universidade Federal do Rio Grande do Sul, Porto Alegre 90050170, RS, Brazil; maitescherers@gmail.com (M.S.d.S.); gicorssac@gmail.com (G.B.C.); carolinedani@yahoo.com.br (C.D.); 2 Departamento de Farmacologia, Instituto de Ciências Básicas da Saúde, Universidade Federal do Rio Grande do Sul, Porto Alegre 90050170, RS, Brazil; bequi.vargas@yahoo.com.br (R.V.A.S.); maiconpmpm@gmail.com (M.P.M.); gabimeirelles@gmail.com (G.M.); saraelisbianchi@gmail.com (S.E.B.); 3 Laboratório de Desenvolvimento Galênico, Faculdade de Farmácia, Universidade Federal do Rio Grande do Sul, Porto Alegre 90610-000, RS, Brazil; valquiria.bassani@ufrgs.br; 4 Curso de Medicina, Universidade de Caxias do Sul, Caxias do Sul 95070-560, RS, Brazil; ltarga@ucs.br; 5 Programa de Pós-Graduação em Ciências Farmacêuticas, Universidade Federal do Rio Grande do Sul, Porto Alegre 90160-093, RS, Brazil; 6 Departamento de Medicina Social, Faculdade de Medicina, Universidade Federal do Rio Grande do Sul, Porto Alegre 90035-003, RS, Brazil; marcelorog@gmail.com

**Keywords:** clinical trials, gastric ulcer, traditional medicine, Southern America

## Abstract

Background/Objectives: The present work aimed to compare the effects of the standardized dry extract from the leaves of *Monteverdia ilicifolia*, popularly known as “espinheira-santa”, with omeprazole in the management of dyspepsia related to gastroesophageal reflux disease (GERD). Methods: A double-blind, randomized, non-inferiority and double-dummy clinical trial was conducted. In total, 86 patients with GERD symptoms were randomized into three groups: Omeprazol (20 mg), *M. ilicifolia* (400 mg), or *M. ilicifolia* (860 mg). Capsules were provided by SUSTENTEC^®^, Pato Bragato, Brazil. It was requested that the participants take three capsules before breakfast and dinner for 4 weeks. Clinical outcomes were obtained at the beginning and end of the study, with GERD symptoms (QS-GERD), the impact of heartburn symptoms on quality of life (HBQOL), and medical records. Results: Overall, 75.6% of the participants showed adherence without any differences among the experimental groups. All groups had significant reductions in both QS-GERD and HBQOL scores. Omeprazole and 400 and 860 mg of *M. ilicifolia* decreased the QS-GERD total scores at the endpoint compared to the baseline (Chi-square = 129.808; *p* < 0.0001), as well as individual item scores, such as heartburn intensity (Chi-square = 93.568, *p* < 0.0001) and heartburn after meals (Chi-square = 126.426, *p* < 0.0001). There were no differences among the experimental groups after the intervention. Conclusions: Our results suggest that capsules with a standardized dry extract from the leaves of *M. ilicifolia* at a dosage of 400 or 860 mg are non-inferior to omeprazole, a proton pump inhibitor.

## 1. Introduction

Gastroesophageal reflux disease (GERD) is highly prevalent in Western countries. In the USA and Europe, almost 20% of adults are affected by GERD, while in Brazil, the prevalence is estimated to be 12% [1]. Beyond the impact of its clinical symptoms, such as heartburn, acid regurgitation, and epigastric pain, GERD impacts the quality of life and well-being of patients. In this context, proton pump inhibitors (PPIs) are widely prescribed, representing the main pharmacological management to minimize GERD effects [2]. However, PPI-induced short- and long-term side effects have raised several concerns, and consequently, new and innovative pharmacological treatments for GERD must be studied [3]. Recently, several medicinal plants have been evaluated for their gastroprotective effects on GERD, including clinical trials [4,5].

In this context, the Brazilian native plant *Monteverdia ilicifolia* Mart. ex Reissek. Biral (basionym: *M. ilicifolia* Mart ex Reissek, Celastraceae), popularly known as “espinheira santa”, has had extensive traditional use in Brazil [6]. Infusions or decoctions are prepared with dry leaves to manage mainly gastrointestinal diseases [7,8]. This species is described in the Brazilian Pharmacopoeia and listed in the National List of Essential Medicines (RENAME) published by the Unified Health System (“Sistema Único de Saúde”, SUS), which contains 12 medicinal herbs [9,10].

Several preclinical studies have indicated the gastroprotective properties of the oral and intraperitoneal administration of water, hexane, and ethyl acetate *M. ilicifolia* extracts in rodent models [11,12,13,14]. The gastroprotective actions of *M. ilicifolia* have been attributed to several phytochemicals, such as phenolic compounds, polysaccharides, and terpenoids [7,15]. Several preclinical studies support the idea that *M. ilicifolia* compounds have multitarget actions that exert different cellular and molecular gastroprotective mechanisms. An important study reported that the dry aqueous extract of *M. ilicifolia* reduced ulceration induced by stress (cold and restraint), with an increase in pH in the gastric contents [16,17]. Interestingly, the intraperitoneal administration of a flavonoid-rich extract of *M. ilicifolia* reduced the gastric lesions induced by ethanol, indomethacin, and acetic acid in rats [18] bringing evidence on the mechanism of action, specifically the antisecretory effects of this extract [18]. In addition, isolated polysaccharides demonstrated a gastroprotective effect in an ethanol-induced gastric damage model [7,19]. These authors suggested that those active polysaccharides of *M. ilicifolia* could act as a mucosal barrier agent [7,19].

In addition to the efficacy approach in the treatment of gastric diseases, the safety of *M. ilicifolia* has raised attention using toxicological studies. The acute and chronic (for 180 days) administration of *M. ilicifolia* leaf aqueous extract did not induce any toxicological effects in rats, mice, or beagle dogs [17] However, a phase I clinical trial showed that this extract, with weekly increment doses of 100 mg up to 2000 mg, was safe and well tolerated [16]. Recently, our group raised in vitro findings of a potential hepatotoxicity [18]. These results were considered to be the eligibility criteria in our project, which was designed to evaluate the efficacy, quality, and safety of *M. ilicifolia* capsules. 

Taken together, our aim is to compare the effects of the standardized dry extract from the leaves of *M. ilicifolia* with a proton pump inhibitor in a double-blind, randomized, non-inferiority and double-dummy clinical trial in the management of dyspepsia related to gastroesophageal reflux disease (GERD).

## 2. Results

### 2.1. Baseline Sociodemographic and Clinical Characteristics

**Figure 1:**
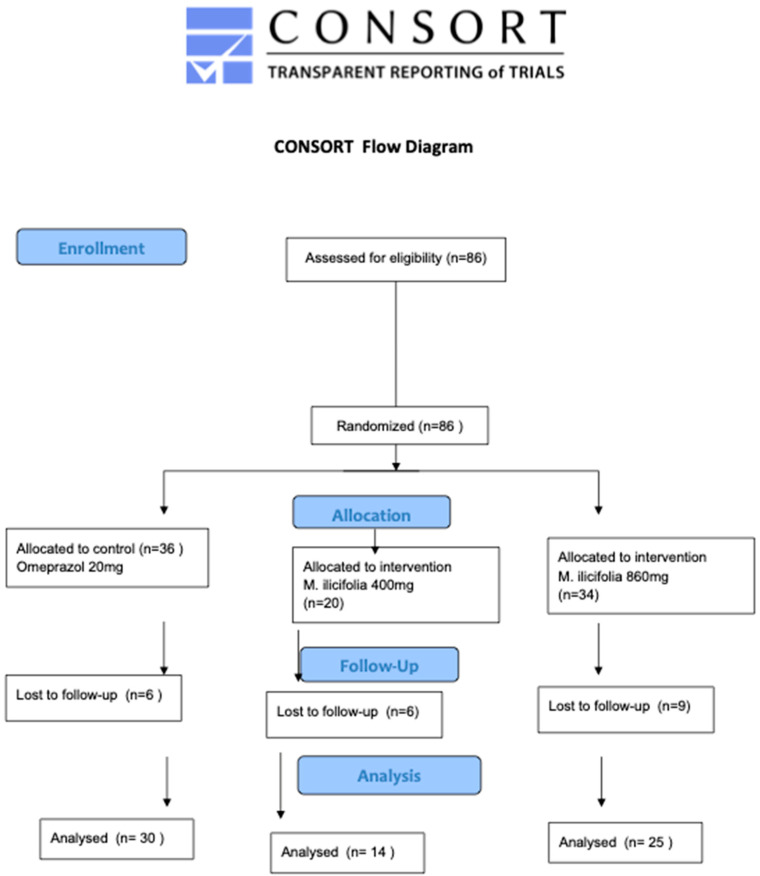
The flow chart of the study.

Out of the 86 patients who were included, 21 individuals were lost at the follow-up (Figure 1); therefore, only 65 patients (75.6%) completed this study. There were no differences among the experimental groups regarding those lost to follow-up [omeprazole (16.67%), *M. ilicifolia* 400 mg (30%), *M. ilicifolia* 860 mg (26.47%) (*p* = 0.956)]. Even with the fact that researchers performed several telephone contacts to request information and feedback, patients did not provide reasons for their loss at the follow-up, except for one patient from the omeprazole group who discontinued due to an adverse effect, specifically nausea.

There were no sociodemographic and clinical differences among the groups, except for age between the *M. ilicifolia* 400 mg and *M. ilicifolia* 860 mg groups (Table 1). The mean age of the individuals who had adherence was 47.2 ± 1.4 years; however, this did not achieve statistical difference in terms of gender distribution.

GERD symptoms and the impact of heartburn symptoms on life quality were evaluated using the QS-GERD and GERD-HBQOL questionnaires, respectively. They were performed at the beginning and at the end of the study. The primary outcome measures are QS-GERD and -HBQOL total scores.

### 2.2. GERD Symptoms

**Figure 2:**
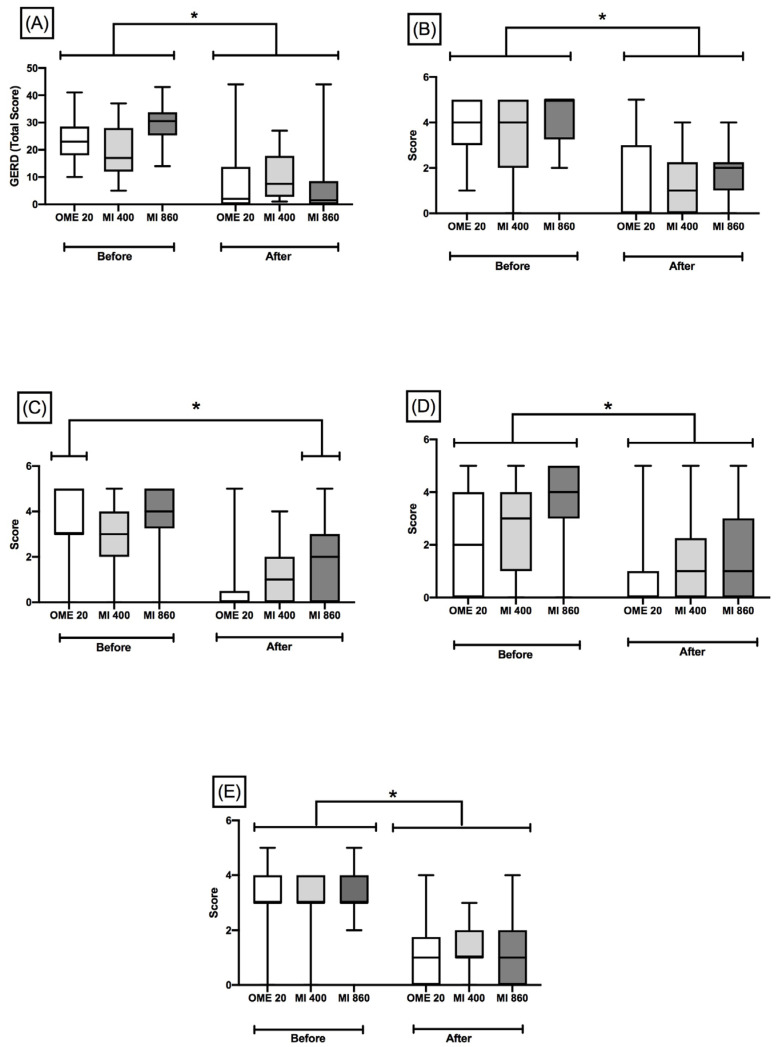
Symptoms of gastroesophageal reflux disease evaluated using QS-GERD questionnaire before and after 4 weeks of intervention with omeprazole or standardized dry extract from leaves of *Monteverdia ilicifolia* (MI). (**A**) Total scores. (**B**) Heartburn intensity. (**C**) Heartburn intensity after meals. (**D**) Regurgitation. (**E**) Degree of dissatisfaction. OME 20: omeprazole 20 mg; MI 400: *M. ilicifolia* 400 mg; MI 860: *M. ilicifolia* 860 mg. Results are expressed as medians (interquartile ranges 25/75). Generalized estimating equations (GEEs), * *p* ≤ 0.05 indicates a significant difference between the baseline and after intervention.

Our preliminary results suggest that capsules with a standardized dry extract from the leaves of *M. ilicifolia* at 400 or 860 mg are non-inferior to omeprazole. The severity of the symptoms of GERD, evaluated by the QS-GERD questionnaire, was reduced by all treatments, since all groups had significantly decreased scores after the 4 weeks compared to the baseline (Figure 2).

Figure 2A shows the QS-GERD total scores, which have a maximum rating of 55, which denotes the most severe symptom intensity. The GEE analyses indicated that the timepoint factor had a strong effect (Chi-square = 129.808; *p* < 0.0001) because all groups had decreased total scores at the endpoint compared to the baseline.

Although the allocation was randomly performed, our preliminary data indicated some significant differences among groups at the baseline, since a significant interaction between timepoint and groups was indicated in the total score of QS-GERD (MI 860, Figure 2A; Chi-square = 10.363; *p* = 0.006). The Sidak test indicated the worst conditions of the higher tested dose of the *M. ilicifolia* group before the treatment period, which can exclude any risk of bias in favor of the intervention group.

Each item of QS-GERD, spanning 0–5, was analyzed, and huge effects of the timepoint factor have been raised by the GEE analyses. The individual scores of all treatments decreased, indicating significant improvements in typical symptoms, such as heartburn intensity (Chi-square = 93.568, *p* < 0.0001; Figure 2B), heartburn after meals (Chi-square = 126.426, *p* < 0.0001; Figure 2C), and regurgitation (Chi-square = 42.436, *p* < 0.0001; Figure 2D). Levels of patient dissatisfaction with the current situation were reduced by all treatments (Figure 2E, Chi-square = 121.51; *p* < 0.0001), without any differences among the positive control group and MI groups before and after treatment for 4 weeks.

### 2.3. Impact of Heartburn Symptoms on Life Quality

**Figure 3:**
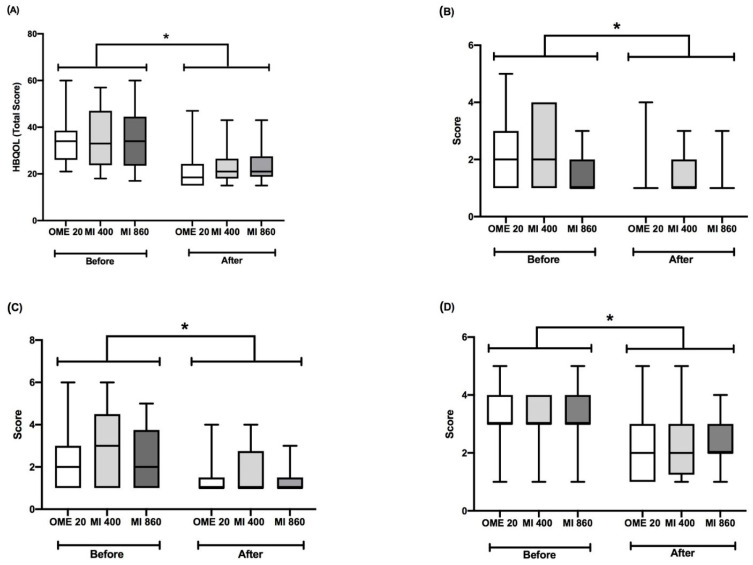
Effects of capsules with omeprazole or a standardized dry extract from leaves of *Monteverdia ilicifolia* at 400 or 860 mg on GERD-induced impairments in daily life and social relations, detected by Heartburn Quality of Life (HBQOL) Questionnaire, before and after 4 weeks of intervention. (**A**) Total scores. (**B**) Impairments in work productivity. (**C**) Impairments in vitality. (**D**) GERD-induced pain. OME 20: omeprazole 20 mg; MI 400: *M. ilicifolia* 400 mg; MI 860: *M. ilicifolia* 860 mg. Results are expressed as medians (interquartile ranges 25/75). Generalized estimating equations (GEEs). * *p* ≤ 0.05 indicates a significant difference between the baseline and after intervention.

The impact of heartburn symptoms on life quality was evaluated by the Heartburn Quality of Life (HBQOL) Questionnaire. The HBQOL scores were improved by capsules with omeprazole or dry extract from leaves of *M. ilicifolia* at 400 or 860 mg (GEE analysis, Figure 3). HBQOL total scores (worst condition rating 69) were significantly reduced by all treatments (Figure 3A; Chi-square = 64.769; *p* < 0.0001).

Each item of the HBQOL was individually evaluated. The omeprazole, *M. ilicifolia* at 400, and *M. ilicifolia* at 860 mg groups after 4 weeks of intervention had improved scores in terms of GERD-induced impairments in work productivity scores; this item ranged from 1 (no time) to 6 (all the time with work impartments) (timepoint effect, Chi-square = 16.459, *p* < 0.001; Figure 3B). The GEE analyses indicated effects of the timepoint factor on GERD-induced impaired vitality, where the scores ranged from 1 to 6, since decreased total scores at the endpoint, compared to the baseline, were found (Chi-square = 31.625; *p* < 0.001; Figure 3C). In addition, capsules with omeprazole or extract from leaves of *M. ilicifolia* at 400 or 860 mg reduced the pain intensity induced by GERD (Chi-square = 35.028; *p* < 0.001; Figure 3D).

## 3. Discussion

The data here reported suggested that capsules with dry extract from leaves of *M. ilicifolia* at both doses, 400 and 860 mg, twice a day, are non-inferior to omeprazole, because the severity of the symptoms of GERD and their impacts on life quality were improved by all interventions.

To the best of our knowledge, this is the first multicenter, randomized, double-blind and controlled clinical trial assessed to compare the effects of *M. ilicifolia* compared to the positive control, omeprazole, bringing about efficacy in terms of the evidence for the decision-making of medicinal plants and herbal medicines available in the Unified Health System (SUS, Brazil).

The randomized double-blind controlled clinical trial here described, indicating that *M. ilicifolia* aqueous extracts at 400 or 860 mg are non-inferior to omeprazole, represents a higher level of evidence to support a recommendation on the use of capsules containing dry extract from leaves of *M. ilicifolia* to treat GERD. In addition, our work supports those preclinical and clinical findings in terms of the safety of *M. ilicifolia* extracts [16,17]. Besides GERD symptoms, our results indicate improvements in quality of life, such as work productivity, sleep, diet, and daily activities as well.

This work has some strengths, since it is a positive-controlled (against omeprazole group), double-blind, randomized, multicenter clinical trial, in which validated questionnaires on GERD symptoms and GERD-related quality of life were used. In addition, we tried to indicate the MI optimal dose with our study design. However, there are some limitations, as follows: the follow-up was only over 4 weeks, and this study had a small sample size. The preliminary findings were obtained with a smaller number of the participants considering sample size determination for a non-inferiority clinical trial, which may have biased the data. In this context, some baseline differences, specifically in terms of GERD symptoms total score as mentioned above, were found, even with proper randomization. However, this baseline difference did not alter the outcome analysis, since it would be in favor of the control group.

Although proton pump inhibitors (PPIs), such as omeprazole, are the first choice for the management of GERD, there are several concerns about their inappropriate prescription, as overuse has been observed and side effects reported, especially those associated with long-term use. Some of them are related to their mechanism of action; gastric hypoacidity, such as changes in bacterial flora; reduction in the absorption of minerals, calcium, iron, and magnesium; enteric infections; hypoacidity-induced hypergastrinemia; and gastric neoplasia. In addition, the involvement of PPIs with a risk of bone fracture, especially hip fracture, and more recently with all-cause dementia has been raised [20,21,22]. In this context, complementary and alternative approaches of treatment, such as herbal medicines, probiotics, and dietary interventions, have also been evaluated [23].

Several medicinal plants have been evaluated in terms of their potential management of GERD, such as *Myrtus communis* L. (Myrtaceae) [5,24]. Rose oil capsules, a traditional Persian medicine, alleviated GERD-induced symptoms in a randomized, placebo-controlled, double-blind clinical trial [25].

In addition to the above-suggested effects, such as antisecretory [18] and as a mucosal barrier agent [7,19] Wonfor et al. (2017) provided in vitro evidence on the anti-inflammatory properties of a hexane extract from *M. ilicifolia* using a human intestinal epithelial cell line [26]. Specifically, *M. ilicifolia* reduced IL-8 secretion induced by a ligand for TLR2 and lipoteichoic acid [27]. In this context, it is important to note that the modulation of the TLRs signaling pathway has been raised as a potential therapeutic approach in several GI diseases [26]; a reduction in gastric inflammation by traditional medicines in gastritis has also been reported, for example, the hydroethanolic extract of *Rosa damascena* showed a strong reduction in IL-8 secretion in *Helicobacter pylori*-infected cells [28] and, as abovementioned, rose oil capsules reduced symptoms in GERD in a clinical trial.

On the other hand, it is possible to postulate that *M. ilicifolia* contains compounds acting on prostaglandin pathways since its aqueous extract fractionated using ethyl acetate had pharmacological effects, specifically hypotension, reverted by indomethacin, an inhibitor of prostaglandin synthase [24] In this sense, misoprostol, a synthetic prostaglandin analog, that can inhibit gastric acid secretion from parietal cells, a mucosal protective agent, induces uterine smooth muscle contractions, inducing labor/abortion. The involvement of prostaglandin pathways as a mechanism of action can be inferred considering that this species was used as a contraceptive and an abortion inductor by women in South America [29]. In this way, pregnant or nursing women, as well as women of reproductive age who were not at that time using any contraceptive method, were considered exclusion criteria in this clinical trial.

Recently, de Paula and colleagues [30,31] showed significant activities of extracts and fractions, including those with aqueous extraction, against *Helicobacter pylori*, as well as the inhibition of urease activity that would allow its survival at acid secretion, and an anti-adhesive effect of hydroalcoholic extracts was detected [32]. *Monteverdia ilicifolia* actions against *H. pylori* can be, at least in part, potential mechanisms of action in clinical benefits on dyspepsia, since it has been preconized that patients suffering with functional dyspepsia (normal endoscopy) who tested positive for *H. pylori* can have some benefits with eradication therapy [30].

Taken together, we can infer that these secondary metabolites, such as phenolic compounds and primary metabolites, including polysaccharides, can be related to *M. ilicifolia*-induced improvements in the clinical GERD symptoms here described as a multitarget strategy based on antisecretory, mucosal barrier, anti-inflammatory, and antibacterial (against *H. pylori*) activities.

In our opinion, beyond the efficacy of the evidence-based validation of decision-making of traditional medicinal plants in the context of our health system (SUS, Brazil), it is relevant to highlight that this broader project raised the potential hepatotoxicity induced by *M. ilicifolia* in a preclinical study, bringing about concerns regarding safety [31]. In the present clinical trial, liver diseases were considered to be exclusion criteria.

## 4. Materials and Methods

### 4.1. Study Design and Ethical Considerations

The preliminary data reported here were collected from December 2021 to May 2023. This multicenter, randomized, double-blind controlled clinical trial was conducted in accordance with good clinical research practice. The research followed the guidelines of the Declaration of Helsinki and Tokyo for humans and was approved by independent Ethics Committees: Comitê de Ética em Pesquisa (CEP)—Hospital de Clínicas de Porto Alegre—HCPA (code number 36540720.1.000.5327); CEP Grupo Hospitalar Conceição (code number 36540720.1.3002.5530); and CEP Prefeitura de Porto Alegre (code number 36540720.1.3004.5338). In addition, this study was registered in the Brazilian Registry of Clinical Trials (ReBEC) under number 10yqwrk6. All participants in this study read and signed a written consent form.

### 4.2. Participants, Criteria, and Randomization

Patients of either gender aged between 18 and 80 years with the presence of gastroesophageal reflux symptoms who asked for clinical care in Basic Health Units in the municipalities of Porto Alegre, Igrejinha, and Nova Petrópolis, located in Rio Grande do Sul State, Brazil (30.0277° S, 51.2287° W, 29.5734° S, 50.7925° W, and 29.3826° S, 51.1186° W, respectively), were potential participants. At this point, the inclusion and exclusion criteria were assessed. Eligible patients were those who claimed to have at least one of the typical GERD symptoms: regurgitation, epigastric pain, or burning pain in the last week prior to the medical appointment. Exclusion criteria were as follows: age <18 or >80 years; intolerance to compounds; pregnant or nursing women; women of reproductive age who were not at that time using any contraceptive method; patients who used any antiulcer or prokinetic drugs during the last week before enrollment; and patients with a history of GERD-related anatomical alterations, such as hiatus hernia, or with a history of liver diseases. Considering that our preliminary preclinical data had indicated a potential interaction of MI compounds with CYP2D6, in order to avoid drug–drug interaction and increase their safety, patients who used chronically CYP2D6 substrates were not included.

Eligible participants were invited; before agreeing to participate, they received information on this study, such as confidentiality, aims, procedures, benefits, and risks. Each participant signed an informed consent form. At this point, 86 participants were enrolled in this study.

Participants were randomly assigned using a virtual platform, www.randomization.com (accessed on 1 September 2024) [33,34,35]. Randomization was stratified by each center. This randomization plan generator system generated tables containing columns and lines, where the left column had an ordered sequence of numbers (the number of participants), and the other column shows the assigned treatments (OME, MI 400, or MI 860). The researchers at each center received the ordered envelopes containing the treatments (and the questionnaires) in accordance with the sequence generated by the randomization platform, and then the patients received their number and respective treatment that was previously organized in accordance with their platform number. Double blinding was ensured using planned interventions, such as the same appearance of capsules in terms of packaging, smell, taste, shape, and color. The allocation remained masked up to the statistical analysis.

### 4.3. Intervention

At this point, 86 participants were randomly assigned to experimental groups: 20 mg omeprazole, 400 mg *M. ilicifolia,* or 860 mg *M. ilicifolia*.

Previously, a double-blind placebo-controlled clinical trial conducted for 28 days indicated that *M. ilicifolia* 400 mg was superior to the placebo group (sugar brown capsules (n = 10), reducing dyspeptic symptoms [6,33]. In addition to 400 mg, we tested those guidelines of national regulatory agencies, specifically ANVISA recommendations, about the *M. ilicifolia* dose (860 mg twice a day) described in the Phytotherapeutic Memento—Brazilian Pharmacopoeia [33].

The technological development aimed to produce a suitable spray-dried powder to be employed as gelatin capsules. The developed technology was transferred to SUSTENTEC^®^, Pato Bragato, Brazil, for scale-up to produce capsules with the standardized dry extract from leaves of *M. ilicifolia* for this clinical trial. The capsules were provided following parameters specified by the Brazilian Health Regulatory Agency (in Portuguese: Agência Nacional de Vigilância Sanitária; RDC 301/2019; RDC 17/2010 and RDC 26/2014). A spray-dried *M. ilicifolia* extract was prepared using a mixture of the excipients starch/colloidal silicon dioxide (Supplementary Materials). The content of chemical markers in the dry extract was 6.5 ± 1.15% and 2.8%, respectively, for total tannins and epicatechin. The fingerprint of the *M. ilicifolia* extract was evaluated by high-performance liquid chromatography (HPLC, Supplementary Figure S1 and Table S1), in accordance with the official plant monograph. In addition to the phytochemical quality control of this extract, microbiological, heavy metal, and pesticides analyses were performed. There was no contamination by heavy metals, pesticides, or mycotoxins. *Salmonella* species and E. coli were not detected. The total mesophilic aerobic bacteria, yeast–mold, and *Enterobacteriaceae* species were below the safe limit for consumption (maximum permissible limits: 10^4^ UFC/g, 10^4^ UFC/g, and 100 NMP/g, respectively).

We registered access to genetic heritage in the National System of Genetic Resource Management and Associated Traditional Knowledge (in Portuguese: Sistema Nacional de Gestão do Patrimônio Genético e do Conhecimento Tradicional Associado—SISGEN/ACE02A1).

It was requested that the participants take three capsules before breakfast and also three capsules before dinner for 4 weeks. The questionnaires were applied (and biological samples were obtained) at the beginning and at the end of the study.

### 4.4. Assessment

The impact of omeprazole or *M. ilicifolia* capsules on GERD symptoms was assessed using the QS-GERD questionnaire, which was translated to Portuguese and validated and comprises 11 items regarding cardinal symptoms of GERD, such as heartburn, regurgitation, sleep disturbance, and pain. The score of each item spans from 0 (best condition) to 5 (worst condition). The primary outcome is QS-GERD total scores, with 55 as the highest score, implying the worst condition.

The HBQOL questionnaire was translated and validated by Pereira et al. (2007) [36] and consists of 12 items about the impact of symptoms related to GERD on quality of life of patients, inducing social restrictions, problems with sleep, loss of work productivity, and symptoms related to eating. For HBQOL total scores, the worst level is indicated by the highest score of 69, and each item was analyzed.

### 4.5. Sample Size and Statistical Analysis

We calculated a sample size of 189 participants (63 for each group) to test non-inferiority in terms of mean among the tested groups, MI 860, MI 400, and omeprazole (with an increase of 10% for possible losses and refusals; this number should be 208). The calculation considered a non-inferiority margin of −5 points on the QS-DRGE scale, power of 80%, a significance level of 5%, a difference of 1 point between the means, and standard deviation equal to 9 points (the data were obtained from the pilot study carried out due to the absence of parameters in the literature). This calculation was performed using the PSS Health online version tool.

Data on sample characterization composition were described as mean ± SD or percentual (%). Analysis included patients who completed the study and the “per-protocol” principle (PP). Normality and homogeneity were evaluated using the Kolmogorov–Smirnov test and Levine test, respectively. To evaluate the main outcomes of this study, a GEE (generalized estimation equation) was performed with gamma distribution followed by the Sidak test. Clinical outcomes are expressed as medians (interquartile ranges 25/75). In the GEEs, it is possible to analyze the impact of isolated factors (group and timepoint) and the interaction between factors. The qualitative variables were analyzed by Chi-square and showed as frequencies. The statistical software IBM SPSS version 20.0 was used, and values of *p* < 0.05 were considered statistically significant.

## 5. Conclusions

The present study showed promising results for the use of capsules containing dry extract from the leaves of *M. ilicifolia* to manage GERD symptoms. Our results add evidence not only for healthcare workers making clinical decisions but also to support regulatory agencies to elaborate on the guidelines of evidence-based phytotherapy. *M. ilicifolia* can be useful to reduce the irrational and widespread use of proton pump inhibitors in the treatment of dyspepsia. Further studies with long-term treatment and a bigger sample size to elucidate the underlying mechanisms may be performed to bring about stronger evidence supporting *M. ilicifolia* as an alternative intervention.

## Figures and Tables

**Table 1:** Baseline sociodemographic and clinical characteristics.

Group Parameters	Omeprazole	MI 400 mg	MI 860 mg	*p* Value
Gender (% n/group)	36 (41.86)	20 (23.26)	30 (34.88)	
Male	24 (66.7)	7 (35)	22 (73.3)	0.17
Female	12 (33.3)	13 (65)	8 (26.7)	
N (%)				
Age (mean ± SE)	46.9 (2.0)	52.5 (2.5) *	43.8 (2.5)	0.05
Weight (Kg) (mean ± SE)	79.0 (3.2)	81.9 (3.6)	77.9 (3.2)	0.75
Height (cm) (mean ± SE)	165.4 (1.4)	167.6 (1.5)	164.3 (1.7)	0.44
Marital status (n-%)	36 (100)	20 (100)	30 (100)	0.21
Single	15 (42.9)	5 (25.5)	13 (43.4)	
Married	16 (42.9)	9 (45)	16 (53.3)	
Divorced	4 (11.4)	4 (20)	1 (3.3)	
Widower	1 (2.9)	2 (10)	0 (0)	
Education (n-%)	36 (100)	20 (100)	30 (100)	0.56
Incomplete elementary school	9 (25)	10 (50)	6 (20)	
Full elementary education	3 (8.3)	1 (5)	4 (13.3)	
Incomplete high school	4 (11.1)	2 (10)	3 (10)	
Complete high school	7 (19.4)	4 (20)	8 (26.7)	
Incomplete higher education	5 (13.9)	0 (0)	4 (13.3)	
Complete higher education	8 (22.2)	3 (15)	5 (16.7)	
Smoker (n -%)	36 (100)	20 (100)	30 (100)	0.53
Never	26 (72.2)	11 (55)	23 (76.7)	
Yes	5 (13.9)	4 (20)	4 (13.3)	
Ex-smoker	5 (13.9)	5 (25)	3 (10)	
Alcohol consumption (n-%)	36 (100)	20 (100)	30 (100)	0.57
No	19 (52.8)	11 (55)	18 (60)	
Yes	17 (47.2)	9 (45)	12 (40)	
Physical exercise (n-%)	36 (100)	20 (100)	30 (100)	0.65
Yes	17 (47.2)	9 (45)	17 (56.7)	
No	19 (52.8)	11 (55)	13 (43.3)	
Symptomatology (n-%)				
Sore throat	7 (19.4)	4 (20)	11 (36.7)	0.22
Dysphonia	11 (30.6)	8 (40)	8 (26.7)	0.60
Globus sensation	19 (52.8)	13 (65)	12 (40)	0.21
Globus pharyngeus	11 (30.6)	9 (45)	10 (33.3)	0.54
Cough	14 (38.9)	10 (50)	15 (50)	0.59

* statistically different (*p* = 0.05) when comparing MI 400 mg vs. MI 860 mg groups.

## Data Availability

The authors declare that the data supporting the findings of this study are available from the corresponding authors upon reasonable request. Information that could compromise the privacy of the research participants is not available.

## Supplementary Materials

The following supporting information can be downloaded at: https://www.mdpi.com/article/10.3390/ph17111559/s1, Figure S1: HPLC fingerprint of *Monteverdia ilicifolia* extract capsules. Two marker components were detected. The details are described in the following Supplement Table S1; Table S1: Chemical components in HPLC of *Monteverdia ilicifolia* capsules.
