# Supplementary material for: Pharmacological Evaluation of the Traditional Brazilian Medicinal Plant Monteverdia ilicifolia in Gastroesophageal Reflux Disease: Preliminary Results of a Randomized Double-Blind Controlled Clinical Trial
Source: Pharmaceuticals (Basel). 2024 Nov 20;17(11):1559. doi: 10.3390/ph17111559 (PMC11597767; doi:10.3390/ph17111559)
Supplement: Supplementary file 1 [file pharmaceuticals-17-01559-s001.zip › pharmaceuticals-3227788-supplementary.pdf]

A spray dried *M. ilicifolia* extract was prepared using a mixture of the excipients starch: colloidal silicon dioxide (92:8) at an excipient:dry residue of the plant ratio of 1:4. The resulting dispersion was spray dried using a o Mini Sray Dryer B-290 (BUCHI®) under the following operating conditions: 10,900 rpm disk rotation rate, 145°C inlet air temperature, 95°C outlet air temperature, and 3 mL/min feed flow and pressure of 2 bar.

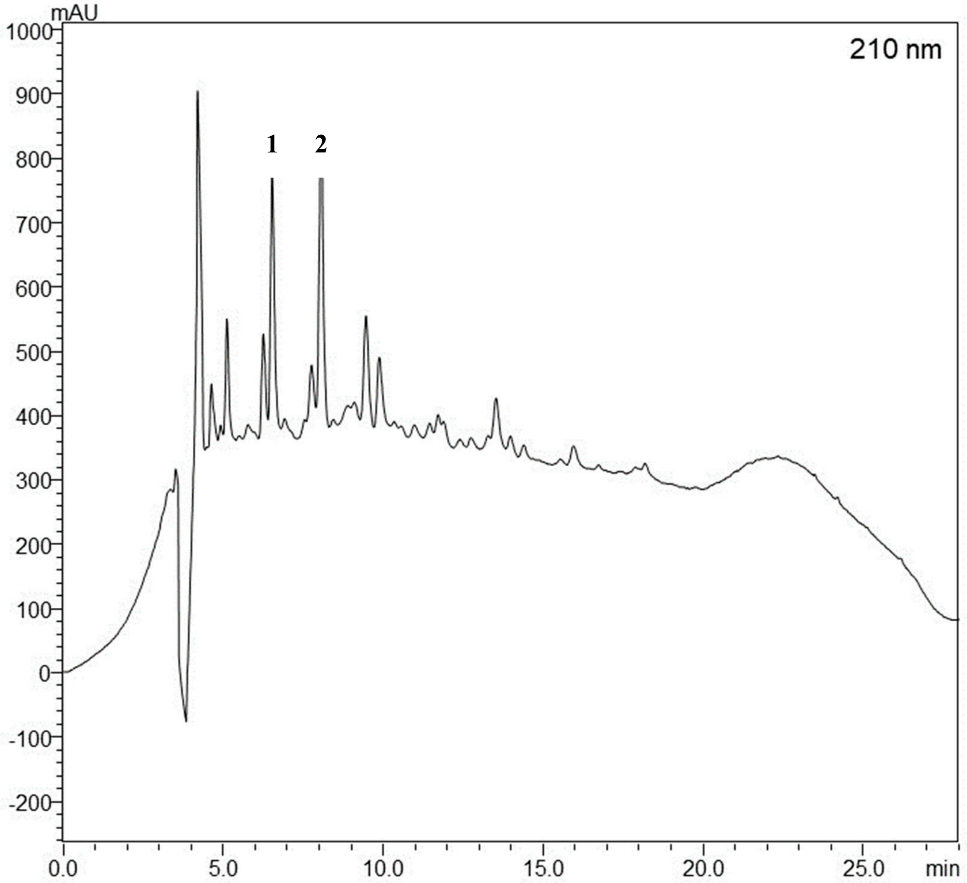

Figure S1. HPLC fingerprint of *Monteverdia ilicifolia* extract capsules. Two marker components were detected. The details are described in the following Supplement Table 1.

Table S1. Chemical components in HPLC of *Monteverdia ilicifolia* capsules

Chromatographic peak	Chemical compound	RT/min
1	catechin	6.514
2	epicatechin	8.042
